# Effects of different ischemic pressures on bar velocity during the bench press exercise: A randomized crossover trial

**DOI:** 10.5114/biolsport.2024.133004

**Published:** 2024-01-02

**Authors:** Dawid Gawel, Jakub Jarosz, Robert Trybulski, Michal Krzysztofik, Piotr Makar, João Guilherme Vieira, Grzegorz Trybek, Michal Wilk

**Affiliations:** 1 Institute of Sport Sciences, Jerzy Kukuczka Academy of Physical Education in Katowice, Poland; 2 Department of Medical Sciences, The Wojciech Korfanty School of Economics, Katowice, Poland; 3 Faculty of Physical Education, Gdansk University of Physical Education and Sport, Gdansk, Poland; 4 Postgraduate Program in Physical Education, Federal University of Juiz de Fora (UFJF), Juiz de Fora, Brazil; 5 Strength Training Research Laboratory, Federal University of Juiz de Fora (UFJF), Brazil; 6 Department of Oral Surgery, Pomeranian Medical University, Szczecin, Poland

**Keywords:** Occlusion, Resistance training, Blood flow restriction therapy, Athletic performance, Power output

## Abstract

The main objective of this study was to evaluate the effects of different ischemic pressures applied during rest intervals on bar velocity during the bench press exercise. 10 resistance-trained males (age = 23.2 ± 2.7 years; body mass = 83.9 ± 9 kg; body height = 181 ± 5.2 cm; bench press 1 repetition maximum (1RM) = 125 ± 16.4 kg; training experience = 5.4 ± 3.4 years) participated in the study. During 4 experimental sessions, following a randomized crossover design, the subjects performed 5 sets of 3 repetitions of the bench press exercise with a load of 60% 1RM under conditions: with ischemia (50% or 80% of arterial occlusion pressure), with SHAM ischemia (20 mmHg) and without ischemia (control condition). For the ischemic conditions cuffs were applied before each set for 6.5 min and released 30 s before the start of the set as reperfusion (6.5 min ischemia + 0.5 min reperfusion). In the control condition, ischemia was not applied. The two-way repeated measures ANOVA showed no significant condition × set interaction for mean bar velocity (MV; *p* = 0.17) and peak bar velocity (PV; *p* = 0.66). There was also no main effect of condition for MV (*p* = 0.58) and PV (*p* = 0.61). The results indicate that ischemic or SHAM treatment (6.5 minutes ischemia or SHAM + 30 s reperfusion) does not affect mean and peak bar velocity during the bench press exercise regardless of the applied pressure.

## INTRODUCTION

Ischemia, also referred to as occlusion or blood flow restriction has been shown to be a training method with the objective of inducing not only chronic adaptations (strength development and muscle hypertrophy), but also acute responses (power output and strength-endurance) [1–4]. Through the application of external compression, most often with the use of pneumatic cuffs the blood flow is partially or fully blocked for designated duration, causing local hypoxia within the muscle tissue. The level of external compression is commonly expressed by % of arterial occlusion pressure (AOP), which is the amount of pressure required to cease the blood flow to a limb, and allows for more precise and individual determination of desired pressure compared to fixed values (e.g. 200 mmHg; [5]). During the resistance training cuffs are typically applied continuously throughout the sets and rest intervals (continuous ischemia [4]), however also other methods have been established, including intermittent ischemia (applied during an exercise [6]), pre-conditioning (ischemia applied before an exercise [7]) and post-conditioning (ischemia applied after the cessation of an exercise [8]).

However, recently a new method of applying ischemia during resistance training has been established. The ischemic intra-conditioning (ischemia applied during the rest periods between exercise) is an innovative but also very attainable, practical, and easy to apply technique [9–13]. In comparison to other methods, it is beneficial on account of its impact on perceptual responses (decreased discomfort). Furthermore, ischemia is applied only during rest intervals, thus possible negative impact associated with frequent use of blood flow restriction (deleterious impact on muscle tissue and attenuated muscle growth directly underneath the cuff) is minimized [13, 14]. However, currently its effect on cardiovascular responses remains unknown, as there is no available data related to this matter. Additionally, the movement structure remains unchanged due to the absence of the cuffs during exercise [15, 16]. Okita et al. [11] reported, that ischemia applied only during the rest intervals may induce a sufficient level of metabolic stress for muscular adaptations. Currently, there are only few studies available related to ischemic intra-conditioning during resistance exercise, yet its results seem encouraging. However, there are several factors requiring clarification, which may impact the effectiveness of this method [1, 12, 17]. Wilk et al. [1] reported acute increases in power output and bar velocity during the bench press exercise with ischemia used during rest intervals [5 sets; 3 repetitions; 60% of one repetition maximum (1RM), 5 minutes ischemia; 80% AOP]. Importantly, such increases were recorded only in sets 3–5, suggesting that the use of ischemia during rest intervals may delay the symptoms of fatigue during successive sets. Interestingly, a similar phenomenon occurred when ischemic intra-conditioning was used on the lower limbs. Trybulski et al. [12] showed that the ischemia used during the rest intervals between sets (Keiser squat; 5 sets; 2 repetitions; 60% 1RM; 4.5 minutes ischemia; 30 s reperfusion; 60% AOP) caused the significantly lower decline in power output in sets 3–5 compared to the control condition. Therefore, those results suggest that ischemic intra-conditioning may counteract increasing fatigue in regard to upper- and lower-body power performance. Importantly, according to Trybulski et al. [12] not only the duration of ischemia, but also the time of reperfusion, referred to as restoration of perfusion and concomitant reoxygenation after previous restriction of blood flow [18] may be of importance in assessing effectiveness of ischemic intra-conditioning. Further, in a different study, Jarosz et al. [17] reported that ischemia (3 minutes rest interval with ~2.5 minutes ischemia and ~30 s cuff inflation and deflation period; 80% AOP), applied during rest intervals between sets increased peak bar velocity at loads 20% 1RM and 50% 1RM during 8 sets of 2 repetitions (10% increase in subsequent set form 20 to 90%1RM) of the bench press exercise. However, increases in bar velocity were not recorded at other loads, suggesting that acute effects of ischemia during rest intervals can be related to the external load used. Surprisingly, Jarosz et al. [17] reported no such increases at the load of 60% 1RM, which is in contrary to the study by Wilk et al. [1].

The available literature regarding ischemic intra-conditioning during resistance exercise is clearly contradictory, furthermore the abundance of modalities impacting the effectiveness of ischemia constitutes a valid necessity for further studies. Given the available research regarding ischemic intra-conditioning during resistance exercise, it has been shown that this method of applying blood flow restriction is effective, however various factors impacting on its efficacy may be differentiated. Furthermore, it is still uncertain which of these modalities are crucial regarding acute changes in power output. Therefore, the aim of this study was to assess the acute effects of different ischemia pressures used during rest intervals between sets of the bench press exercise on bar velocity changes. Given that the bench press is one of the most frequently used upper-body exercises, and it was previously used in research regarding this matter it was selected in this study [1, 19, 20]. To the best of our knowledge there was no other study analyzing the acute effects of different cuff pressures used during intra-conditioning ischemia. It has been shown that when the cuff pressure is set to 60% AOP or 80% AOP, ischemic intra-conditioning has a positive impact on bar velocity and power output [1, 12, 17]. However, previous research did not investigate the impact of cuff pressures < 60% AOP. Furthermore, given that it has been previously recommended that research regarding ischemia should include SHAM or placebo group [7], cuff pressure values of 50% AOP, 80% AOP and 20 mmHg (SHAM ischemia) have been selected. We hypothesized such outcome would occur only when the higher pressures were applied (50% AOP, 80% AOP).

## MATERIALS AND METHODS

### Experimental Approach to the Problem

The experiment was carried out according to a randomized crossover design. All subjects participated in 4 different testing protocols (2 ischemia conditions, 1 SHAM ischemia condition (SHAM) and 1 control condition) performed in counterbalanced order, arranged 4 to 7 days as washout period. The experiment was preceded by 2 familiarization sessions two weeks before the main testing sessions and 1RM strength test performed one week before the main testing sessions. During each experimental sessions the participants performed 5 sets of 3 repetitions of the bench press exercise with a load of 60%1RM [1]. In order to analyze the impact of cuff pressure on bar velocity during ischemic conditions 2 different values of cuff pressure were applied: ~50%AOP, ~80%AOP, however for the SHAM condition 20 mmHg pressure was used. Ischemia was not used during the control condition. The rest intervals between sets lasted 7 minutes and ischemia or SHAM were applied for 6.5 minutes (6.5 minutes ischemia or SHAM + 30 s reperfusion), excluding control condition. The peak and mean bar velocity were measured using a linear position transducer. All testing sessions were performed in the Strength and Power Laboratory at the Academy of Physical Education in Katowice, Poland. The experimental procedure did not change at any stage of the experiment.

### Subjects

Ten healthy resistance-trained males (age = 23.2 ± 2.7 years; body mass = 83.9 ± 9 kg; body height = 181 ± 5.2 cm; bench press 1RM = 125 ± 16.4 kg; training experience = 5.4 ± 3.4 years) volunteered for the study. The inclusion criteria were: a) free from neuromuscular and musculoskeletal disorders, b) at least 2 years of resistance training experience, c) bench press 1RM performance of at least 120% body mass (verified during 1RM strength test), d) free of cardiovascular disease, including arterial hypertension, atrial fibrillation, thrombosis, myocardial insufficiency (self-declaration). Before providing their written informed consent, the participants were briefed about the potential risks of the study, moreover they were allowed to withdraw from the study at any time. They were also instructed to maintain their dietary and sleep habits throughout the course of the experiment. The randomization was performed with an online generator (randomization. org). Each participant received a number and sequence of the sessions. The participants were not informed about the expected outcomes of the experiment. The study protocol was approved by the Bioethics Committee for Scientific Research, at the Academy of Physical Education in Katowice, Poland (2/2019), and all procedures were in accordance with the ethical standards of the Declaration of Helsinki, 1983.

### Procedures

#### Familiarization Session

Two weeks before the main experiment the subjects performed two familiarization sessions, arranged 2 to 4 days apart. During the familiarization sessions, after performing a standardized warm-up consistent with the subject’s normal training habits each subject performed 5 sets of 3 repetitions of the bench press exercise with a load of 60% of their estimated 1RM with ischemia (50% AOP during the first session, and 80% AOP during the second session) used before the first set and during the rest intervals between sets [1]. The familiarization sessions were performed in order to minimize possible learning effects during the main testing sessions.

### 1RM Strength Test

One-week before the main experiment the free-weight bench press 1RM test was carried out. After arrival, the subjects cycled on a cycle-ergometer for 5 minutes, at an exercise intensity that induced a heart rate of~130 bpm, followed by a general upper-body warm-up as described elsewhere [21]. Then, the study participants performed 15, 10, 5, 3 bench press repetitions using 20, 40, 60 and 80% of their estimated 1RM, respectively. Then the load was increased by 2.5 to 10 kg for each subsequent attempt [22]. This process was repeated until failure (within a maximum of five attempts). The rest interval between successful trials was 5 minutes. Hand placement on the barbell, as well as movement tempo during the 1RM test were volitional. Testing was performed using an Olympic barbell (20 kg, 2.8 cm diameter, 1.92 m length) (Eleiko International, Halmstad, Sweden).

### Experimental Sessions

The experimental procedure was designed similarly to that previously described in a different study regarding ischemic intra-conditioning [1], in order to allow for a comparison of the results. During each experimental session subjects performed 5 sets of 3 repetitions of the bench press exercise with a load of 60% 1RM with maximal possible velocity in the eccentric and concentric phase of the movement, moreover a 7-minutes rest interval between sets was used. For the control condition ischemia was not applied, however during other experimental sessions ischemia or SHAM was applied before the first set and during every rest interval between sets. Under ischemic conditions the cuff pressure was set to ~50%AOP or ~80%AOP, however in SHAM to 20 mmHg. A linear position transducer (Tendo Power Analyzer, Tendo Sport Machines, Trencin, Slovakia) was utilized to measure peak and mean bar velocity. It has been shown that for the measurement of bar velocity changes during the free-weight bench press exercise at 60% 1RM linear position transducers show good reliability; intra-class correlation co-efficient (ICC) 0.81, standard error of measurement percentage (SEM%) 5.1% for peak bar velocity and ICC 0.83, SEM% 5,8% for mean bar velocity [23, 24]. Peak bar velocity was obtained from the best repetition performed in each set. Mean bar velocity was obtained as the mean of 3 repetitions performed in each set. Tendo Power Analyzer unit cord was attached to the end of the barbell via Velcro. The device was placed in a manner that allowed for the most perpendicular trajectory of the cord. The same experienced investigator was responsible for accuracy of each measurement performed with the Tendo Power Analyzer unit during the course of the study.

### Ischemic procedure

For the ischemic and SHAM conditions pressure cuffs were applied bilaterally as high as possible in the close proximity to axillary fossa. This study utilized Fit Cuffs (Fit Cuffs ApS, Denmark), which are 7-cm wide. For ischemic (~50%AOP, ~80%AOP) and SHAM (20 mmHg) conditions, before each set ischemia or SHAM were applied for 6.5 minutes and released 30 s before the beginning of a set (6.5 minutes ischemia + 30 seconds reperfusion). In order to determine AOP of each participant (~50% or ~80% AOP conditions) after the completion of general warm-up and a 5-minute rest interval the value of full arterial occlusion pressure (100% AOP) was determined (subjects remained in a seated position). A handheld Edan SD3 Doppler with an OLED screen and a 2 mHz probe made by Edan Instruments (Shenzhen, China) was utilized [1, 17]. The measurement was conducted on the radial artery, twice on each limb and the measurements were 5 min apart in the subject [3]. In case the obtained differences were within 20 mmHg, the mean of the two measurements was used to set the cuff pressure for the exercise protocol [1]. During ischemic conditions cuff pressure was set to ~50% AOP (78 ± 9 mmHg) or ~80% AOP (125 ± 13 mmHg), and during the SHAM condition to 20 mm Hg.

### Statistical Analysis

All statistical analyzes were performed using Statistica 9.1. Results are presented as means and standard deviations. The Shapiro-Wilk test was used to verify Gaussian distribution, homogeneity, and sphericity of the sample data variances, respectively. Differences between the conditions were examined using two-way repeated measures ANOVA [4 conditions (ischemia at 80% AOP vs. ischemia at 50% AOP vs. vs. SHAM vs. control) × 5 sets of bench press]. Effect sizes (ES) for main effects and interactions were determined by partial eta squared (η2). Partial eta squared values were classified as small (0.01–0.059), moderate (0.06–0.137) and large (> 0.137). Post hoc comparisons using Tukey’s test were conducted to locate the differences between mean values when the main effect or an interaction was found. For pairwise comparisons, ESs were determined by Cohen’s d which was characterized as large (d > 0.8), moderate (d between 0.8 and 0.5), small (d between 0.49 and 0.20), and trivial (d < 0.2). Percent changes with 95% confidence intervals (95CI) were also calculated. Statistical significance was set at p < 0.05.

## RESULTS

The two-way repeated measures ANOVA did not show statistically significant condition × set interaction for MV (conditions × sets; *p* = 0.17; η^2^ = 0.13; Table 1) as well as no statistically significant condition × set interaction for PV (conditions × sets; *p* = 0.66; η^2^ = 0.08; Table 2). There was also no main effect of condition for MV (*p* = 0.58; η^2^ = 0.07) and PV (*p* = 0.61; η^2^ = 0.06). The effect size comparison between the experimental conditions for all measured variables is presented in Table 3.

**TABLE 1:** Mean bar velocity during five sets of the bench press exercise under four experimental conditions.

Condition	Set 1 MV [m/s] (95%CI)	Set 2 MV [m/s] (95%CI)	Set 3 MV [m/s] (95%CI)	Set 4 MV [m/s] (95%CI)	Set 5 MV [m/s] (95%CI)	*p* for interaction	*p* for main effect of condition
Control	0.73 ± 0.11 (0.54 to 0.97)	0.72 ± 0.11 (0.53 to 0.94)	0.69 ± 0.09 (0.54 to 0.85)	0.70 ± 0.11 (0.51 to 0.92)	0.72 ± 0.09 (0.59 to 0.91)	0.17	0.58

SHAM	0.70 ± 0.10 (0.56 to 0.86)	0.71 ± 0.09 (0.57 to 0.84)	0.72 ± 0.12 (0.56 to 0.94)	0.69 ± 0.09 (0.52 to 0.81)	0.70 ± 0.12 (0.55 to 0.93)		

50% AOP	0.67 ± 0.09 (0.52 to 0.82)	0.67 ± 0.08 (0.58 to 0.77)	0.70 ± 0.05 (0.60 to 0.77)	0.70 ± 0.07 (0.59 to 0.82)	0.69 ± 0.06 (0.57 to 0.79)		

80% AOP	0.70 ± 0.10 (0.61 to 0.92)	0.68 ± 0.11 (0.50 to 0.86)	0.71 ± 0.15 (0.44 to 0.99)	0.69 ± 0.14 (0.44 to 0.95)	0.69 ± 0.14 (0.45 to 0.89)		

Note: All data are presented as mean with standard deviation while 95% confidence intervals (CI) are presented in parentheses. MV = mean bar velocity; AOP = arterial occlusion pressure; SHAM = sham ischemia.

**TABLE 2:** Peak bar velocity during five sets of the bench press exercise under four experimental conditions.

Condition	Set 1 MV [m/s] (95%CI)	Set 2 MV [m/s] (95%CI)	Set 3 MV [m/s] (95%CI)	Set 4 MV [m/s] (95%CI)	Set 5 MV [m/s] (95%CI)	*p* for interaction	*p* for main effect of condition
CONTROL	1.03 ± 0.17 (0.90 to 1.15)	1.01 ± 0.15 (0.90 to 1.11)	0.99 ± 0.13 (0.90 to 1.09)	0.98 ± 0.15 (0.88 to 1.09)	1.01 ± 0.13 (0.91 to 1.10)	0.66	0.61

SHAM	1.02 ± 0.15 (0.91 to 1.12)	0.98 ± 0.15 (0.87 to 1.08)	0.99 ± 0.17 (0.87 to 1.11)	0.99 ± 0.15 (0.88 to 1.09)	0.98 ± 0.17 (0.86 to 1.10)		

50% AOP	0.97 ± 0.14 (0.87 to 1.07)	0.97 ± 0.16 (0.85 to 1.08)	0.97 ± 0.12 (0.88 to 1.05)	1.00 ± 0.15 (0.89 to 1.11)	0.97 ± 0.10 (0.90 to 1.04)		

80% AOP	1.00 ± 0.14 (0.90 to 1.09)	0.97 ± 0.17 (0.85 to 1.09)	1.00 ± 0.20 (0.85 to 1.14)	0.97 ± 0.19 (0.83 to 1.11)	0.96 ± 0.19 (0.83 to 1.10)		

Note: All data are presented as mean with standard deviation while 95% confidence intervals (CI) are presented in parentheses. PV = peak bar velocity; AOP = arterial occlusion pressure; SHAM = sham ischemia.

**TABLE 3:** Differences in effect size between experimental conditions.

Comparision	Set 1		Set 2		Set 3		Set 4		Set 5	

	MV	PV	MV	PV	MV	PV	MV	PV	MV	PV
CONTROL vs SHAM	0.29	0.06	0.1	0.2	0.28	0	0.1	0.07	0.18	0.2
CONTROL vs. 50%AOP	0.6	0.39	0.52	0.26	0.14	0.16	0	0.13	0.38	0.34
CONTROL vs. 80%AOP	0.29	0.19	0.36	0.25	0.16	0.06	0.08	0.06	0.24	0.31
SHAM vs. 50%AOP	0.32	0.34	0.47	0.06	0.22	0.14	0.12	0.07	0.11	0.07
SHAM vs. 80%AOP	0	0.14	0.3	0.06	0.07	0.05	0	0.12	0.08	0.11
50%AOP vs. 80%AOP	0.32	0.21	0.1	0	0.09	0.18	0.09	0.18	0	0.07

Note: MV = mean bar velocity; PV = peak bar velocity; AOP = arterial occlusion pressure; SHAM = sham ischemia.

## DISCUSSION

The main finding of this study was that ischemia or SHAM applied during rest intervals between sets does not induce changes in mean and peak bar velocity during multiple-set of the bench press exercise at 60% 1 RM. The lack of significant differences occurred in both ischemic conditions (~50%AOP, ~80%AOP) and SHAM condition (20 mmHg). Therefore, it might be concluded that neither different cuff pressures nor SHAM influenced acute power performance changes, which is in contrast to our initial hypothesis.

The result of this experiment is in contradiction to previous research related to the acute impact of ischemic intra-conditioning on power performance, however there are several differences between presented and previous study protocols that should be considered [1, 12, 17]. Currently only a few studies investigated the acute effects of ischemic intra-conditioning during resistance exercise on power output or bar velocity changes and reported its positive influence. In one study, which assessed the effects of ischemic pressure (80% AOP) applied during rest intervals between sets of the bench press exercise at 60% 1RM significant increases in power output and bar velocity were recorded [1]. Further, Trybulski et al. [12] reported a significantly lower decline in power output when ischemia was applied between sets of the Keiser squat exercise (60% 1RM; 60% AOP). Finally, the study by Jarosz et al. [17] showed increases in peak bar velocity, but only at loads 20% 1RM and 50% 1RM when ischemia (80% AOP) was applied during rest intervals between 8 sets of 2 repetitions of the bench press exercise (10% increase in subsequent set from 20 to 90% 1RM). First of all, the significance of cuff pressure should be considered. The efficacy of higher cuff pressures regarding the acute impact on power output has been established in previous studies. Gepfert et al. [25] found that the use of extremely high cuff pressure (150% AOP) during the squat exercise (70% 1RM) resulted in significant increases in bar velocity and power output, however such result was not observed when the cuff pressure was set to 100% AOP. Similarly, for the upper-body significant increases in the number of repetitions performed, time under tension and 1RM performance were reported during the bench press exercise when cuffs with pressure value equal to 150% AOP were applied, however the 100% AOP pressure was not sufficient to induce such changes [26]. Nonetheless, these finding are related to ischemia used during resistance exercise and high or extremely high cuff pressures, not examined in our study, which most likely would not be possible to tolerate for the duration of the rest interval (6.5 minutes). Interestingly, previous research related to the impact of cuff pressure on blood flow responses [27, 28] exhibited a not-linear response between cuff pressure and blood flow (blood flow is reduced to approximately the same degree regardless of cuff pressure). Crossley et al. [27] showed that when the cuff is applied at rest ischemic interventions with pressures ranging from 40–80% AOP elicit very similar blood flow responses (measured by Doppler Ultrasound). Further, Mouser et al. [28] reported that pressures from 40% to 90% AOP decrease blood flow to a similar degree. Correspondingly, recent study by Hornikel et al. [29] determined that the 50% AOP is a minimal threshold pressure required to elicit a significant decrease in arterial blood flow in lower-limbs. However, the aforementioned studies are related to measurements performed at rest, therefore the response during exercise may be different. On the other hand, studies comparing different cuff pressures during maximal voluntary contractions (isometric knee extension) showed decreased torque only when higher (60% and 80% AOP) pressures were used compared to lower (40% AOP) pressures [30, 31]. Although, no differences between used cuff pressures occurred in the presented study, the significance of other modalities (subsequently discussed) related to ischemic intra-conditioning should also be considered. Most notably, given that the same cuff pressure (~80% AOP) was used, as in the other study related to this issue, in which significant increases in power output and bar velocity were reported [1].

Further, among other modalities, the impact of the duration of ischemia should be recognized as well. In one study, which assessed the effects of ischemic pressure applied during rest intervals between sets of the bench press exercise at 60% 1RM significant increases in power output and bar velocity were recorded [1]. Although, in the presented study the same external load (60% 1RM), and the same cuff pressure (~80% AOP) was used, we did not observe increases in peak or mean bar velocity. Such discrepancies might be explained by distinct durations of ischemia which in this study was longer compared to the study by Wilk et al. [1] (6.5 vs. 5 minutes, respectively). Furthermore, our study, contrary to the previous research by Wilk et al. [1] utilized a reperfusion period of 30 s following ischemia, which relates to the time between cessation of blood flow restriction and the commencement of exercise. Therefore, the duration of ischemia, as well as the time of the reperfusion period might play an important role in effectiveness of ischemic interventions during rest intervals between sets of resistance exercise, as previously suggested by Trybulski et al. [12]. However, to the best of the authors knowledge there is no study available comparing the direct impact of different durations of reperfusion time following ischemia on exercise performance. Jarosz et al. [17], similarly to the presented study also reported no increases in bar velocity during the bench press exercise against a load of 60% 1RM, however showed increases in peak bar velocity at 20% 1RM and at 50% 1RM. In this study the duration of ischemia during rest intervals between sets significantly differed compared to the presented study (2.5 vs. 6.5 minutes, respectively). Therefore, it seems that the longer (6.5 minutes) duration of ischemia used between sets of resistance exercise (as used in the presented study) is inferior compared to shorter durations (2.5–5 minutes). Further, it might be concluded that the leading outcomes regarding acute increases in power output performance appear to occur when the ischemia lasts ~5 minutes [1, 12]. Moreover, a clinical study by Ghosh et al. [32] found that only 4 minutes of ischemia is sufficient to reach the threshold for ischemic stimulus in humans. Furthermore, a recent study by Salagas et al. [33] found that a single cycle of short duration (5 minutes) ischemic pre-conditioning increased mean bar velocity and the number of repetitions performed during repeated sets of the bench press exercise. Given that, it might be concluded that the durations of ischemia applied between sets during resistance exercise which are longer than 5 minutes may not be beneficial for acute changes in power output performance, however currently there is no available data regarding shorter durations of ischemia with the use of different cuff pressures. Therefore, for ischemic intra-conditioning, the importance of ischemic duration appears to be superior to cuff pressure, given that both studies mentioned above [1, 17] utilized cuff pressures equal to ~80% AOP, similar to that which was used in our study.

In regard to ischemic intra-conditioning aforementioned factors, including cuff pressures, the duration of ischemia, and the duration of reperfusion seem to be of importance. However, also other factors such as various training variables and the type of exercise (upper- and lower-body) should be taken into consideration. Furthermore, it has been proposed that the acute impact of ischemia may also be related to the characteristics of the occluded limb, thus its circumference [3, 34, 35], length [25], and composition [36]. Therefore, most likely there are numerous factors impacting on the effectiveness of ischemia, however currently there is no sufficient scientific data to corroborate. It should be noted, that despite the fact that no significant increases in mean or peak bar velocity were observed when ischemia or SHAM ischemia were applied, the power performance was not decreased. Moreover, ischemia may induce different physiological responses, which regrettably are beyond the scope of this study. It has been suggested that ischemic intra-conditioning may have a beneficial effect on acute metabolic and hormonal responses [9, 13]. Teixeira et al. (2018) reported higher metabolic stress (accounted for by blood lactate) when ischemia was applied during rest intervals compared to conditions without ischemia and intermittent ischemia, during high-load resistance exercise (3 sets of 8 repetitions; knee extension at 70% 1RM). The mechanism of ischemia used between sets related to improved exercise performance, similarly to ischemic pre-conditioning might be regulated by metabolic and vascular pathways; neuronal, humoral, and systemic responses [7, 37]. Ischemia has been shown to positively impact de-oxygenation of muscular Hb/Mb, absorption kinetics of pulmonary O_2_, muscle vasodilatation and opening of the ATP-dependent K^+^ channels by increasing the energy stocks after ischemia [12, 37, 38]. Moreover, according to Torma et al. [39] ischemic intra-conditioning may impact on the gene expression of angiogenesis and mitochondrial biogenesis, thus affecting the time of muscle repair and muscle hypertrophy, which may be of importance not regarding acute, but chronic responses following ischemia used during rest intervals between sets. However, regrettably this was not evaluated in the presented research, therefore composing a limitation of this study. Thus, this issue, as well as previously mentioned factors such as the duration of ischemia and reperfusion [12] should be addressed in further research regarding ischemic intra-conditioning. Moreover, given that our study similarly to other research related to this topic [1, 17] included resistance-trained subjects (bench press 1 RM exceeding body weight), it remains unclear whether or not this method may be beneficial only for elite, but also recreationally trained or untrained individuals. Interestingly, a systematic review by Incognito et al. [40] showed that recreationally active population is more responsive to ischemic treatment before an exercise compared to trained and highly trained population, therefore this issue should also be further explored.

## CONCLUSIONS

It is indicated by the results of this study that ischemic or SHAM treatment (6.5 min ischemia or SHAM + 30 s reperfusion) used during rest intervals between sets does not acutely influence mean and peak bar velocity, regardless of the value of applied pressure. Therefore, we believe future studies should investigate different durations of ischemia during rest intervals between sets, as well as reperfusion period. Further, the power performance was not decreased and the ischemic intra-conditioning intervention may potentially increase physiological responses and therefore mediate positive muscle adaptation not investigated in this study. However, taking into consideration the results of this study, as well as previous research related to this method, it is still not possible to determine which factors are crucial in order to achieve increased power performance following ischemic intra-conditioning.
